# Isolated Aortic Valve Replacement Versus Concomitant Replacement of the Ascending Aorta and Aortic Valve: A Statistical Analysis and Literature Review

**DOI:** 10.7759/cureus.88247

**Published:** 2025-07-18

**Authors:** Selman Dumani, Devis Pellumbi, Ermal Likaj, Laureta Dibra, Edlira Rruci, Saimir Kuci, Stavri Llazo, Alessia Mehmeti, Ali Refatllari, Altin Veshti

**Affiliations:** 1 Division of Cardiac Surgery, University Hospital Center “Mother Theresa”, Tirana, ALB; 2 Division of Cardiac Surgery, University Hospital Center "Mother Theresa", Tirana, ALB; 3 Division of Cardiology, University Hospital Center "Mother Theresa", Tirana, ALB; 4 Division of Anaesthesiology, University Hospital Center “Mother Theresa”, Tirana, ALB

**Keywords:** aortic valve, aortic valve replacement, ascending aorta, ascending aorta replacement, p-value, simultaneous surgery

## Abstract

Introduction: In contemporary practice, a cardiac surgeon is often confronted with critical questions. Should the ascending aorta be replaced concomitantly when the aortic valve is replaced? Does simultaneous surgery significantly increase operative risk? Although the literature offers answers, the decision for simultaneous surgery remains individualized for every patient, taking into account clinical and nonclinical data. Concomitant replacement of the ascending aorta and aortic valve was first introduced in the 1960s, and since then, the debate has continued regarding the most appropriate surgical strategy for managing aneurysmal disease of the ascending aorta and the criteria guiding such interventions. We aim to present and compare our institutional data on isolated aortic valve surgery versus concomitant replacement of the aortic valve and ascending aorta and to compare our findings with the literature.

Methods: We retrospectively analyzed our database of adult patients (≥18 years) who underwent isolated aortic valve replacement (AVR) or concomitant AVR and ascending-aorta replacement from 2007 to 2023 at the Cardiac Surgery Service, University Hospital Center “Mother Teresa,” Tirana. Data were extracted from operating-room registers and medical records. Demographic, pre-, intra-, and postoperative clinical variables were collected. Continuous variables are presented as mean ± standard deviation; categorical variables as percentages. Statistical analysis was performed with IBM SPSS Statistics for Windows, version 26.0.

Results: The study included 491 patients who underwent isolated AVR and 131 patients who had concomitant AVR with ascending-aorta replacement. Sex (p < 0.001) and age (p < 0.001) differed significantly between the groups. Concomitant surgery involved 102 men (77.9%), whereas isolated surgery involved 311 men (63.3%). The mean age for isolated AVR was 62.28 ± 10.76 years; for concomitant surgery, 57.33 ± 11.90 years. New York Heart Association (NYHA) class was also significant, with 457 patients (94.8%) in NYHA II-III in the isolated group and 128 patients (97.7%) in the concomitant group. Arterial hypertension and diabetes mellitus were statistically significant comorbidities; renal insufficiency, smoking, obesity, and COPD were not. Cardiopulmonary bypass (CPB) time (83.85 ± 22.63 min vs. 111.09 ± 23.67 min; P < 0.001) and aortic cross-clamp time (65.22 ± 19.20 min vs. 89.56 ± 20.71 min; P < 0.001) differed significantly. Aortic annulus diameter, body-surface area, and indexed effective orifice area (SEPi) were also significant variables. Although hospital mortality was not statistically different, it was the most clinically relevant outcome in comparison with several international centers, underscoring the good results of our surgical team. Hospital mortality for isolated AVR was 1.6% (eight patients); for concomitant surgery, 2.29% (three patients). This analysis was limited to in-hospital outcomes. No long-term follow-up data, such as reoperation rates, late complications, or survival beyond discharge, were included.

Conclusion: Concomitant replacement of the aortic valve and ascending aorta markedly prolongs CPB and aortic cross-clamp times compared with isolated AVR, yet mortality remained low and comparable with leading international centers. Although concomitant surgery carries a higher operative risk than isolated AVR, our experience demonstrates that it can be performed with satisfactory outcomes.

## Introduction

In isolated aortic valve replacement or concomitant surgery of the aortic valve and ascending aorta, while most patients present clear indications for one or the other, in daily practice, surgeons encounter borderline cases. Should the ascending aorta be addressed? Does concomitant surgery substantially raise operative risk? Which intra- and postoperative variables prove statistically significant? Wheat et al. first described concomitant replacement of the aortic valve and ascending aorta in 1964 [1], and the optimal surgical strategy for an aneurysmal ascending aorta - and the indications for intervention - have been debated ever since. Techniques described include aortic wrapping [2], aortoplasty [3], plication [4], and prosthetic graft replacement [5]. These are the techniques used in the cases of repair or replacement of the ascending aorta, and newer remodeling or reimplantation techniques were not considered. However, the effect of concomitant ascending-aorta replacement, in line with current guidelines [6-8], on perioperative morbidity and mortality remains sparsely documented. A substantial proportion of patients undergoing AVR exhibit a dilated ascending aorta [9]. An untreated aneurysm may lead to catastrophic events such as dissection or rupture [10,11]; therefore, guidelines recommend concomitant replacement when the ascending aorta diameter exceeds 45 mm, particularly in bicuspid-aortic-valve patients [12,13]. Because AVR alone corrects hemodynamic anomalies but not intrinsic aortopathy, replacing the ascending aorta during AVR is considered a valid option despite the added risk. Concomitant surgery has, accordingly, become routine practice at our center.

Nowadays, large-scale studies on concomitant surgery are rare in the literature, thereby increasing the need for studies with large patient populations, not only to reflect operative outcomes but also to demonstrate long-term results during follow-up. Despite the fact that global centers have demonstrated their results on concomitant surgery of the aortic valve and ascending aorta versus isolated aortic surgery, in our country, it was necessary to provide an overview of our own results with the aim of understanding current outcomes, the experience gained over the years, and implementing measures to improve these results.

## Materials and methods

Study design and patient selection

This is a retrospective, observational, single-center study that included adult patients (aged ≥18 years) who underwent cardiac surgery at the Cardiac Surgery Service, University Hospital Center “Mother Teresa,” Tirana, between January 2007 and December 2023. In our study, 131 patients who underwent concomitant surgery and 491 patients who underwent isolated aortic valve surgery were analyzed. The number of patients in this study was satisfactory, and the data were nearly complete, with only minor exclusions due to missing information. The aim was to compare clinical outcomes between patients undergoing isolated AVR and those undergoing concomitant replacement of the aortic valve and ascending aorta (AVR+AAR).

Inclusion Criteria 

Patients ≥18 years of age who underwent either isolated AVR or AVR+AAR due to aortic valve pathology (stenosis, insufficiency, or mixed lesion), with or without ascending aortic aneurysm, were included in the study. All patients had complete preoperative, intraoperative, and postoperative data available in the hospital registry.

Exclusion Criteria 

Patients were excluded if they had (i) active infective endocarditis, (ii) Stanford type A aortic dissection, (iii) previous cardiac surgery (redo operations), or (iv) undergone Bentall procedures or any root replacement involving coronary reimplantation. The decision for isolated surgery or concomitant surgery was personalized for each patient, primarily based on guideline recommendations. The measurements adhered to the American Society of Echocardiography (ASE)/European Association of Cardiovascular Imaging (EACVI) guidelines and were performed by accredited personnel. 

Data collection

Data were obtained from operative room logs, hospital electronic records, echocardiographic reports, and discharge summaries. Demographic, preoperative, intraoperative, and postoperative clinical parameters were recorded.

Preoperative variables included age, sex, body surface area (BSA), comorbidities (arterial hypertension, diabetes mellitus, chronic kidney disease, chronic obstructive pulmonary disease, obesity, peripheral vascular disease, and smoking), and functional classification according to the New York Heart Association (NYHA).

Intraoperative parameters included cardiopulmonary bypass (CPB) time, aortic cross-clamp time, and the type of prosthetic valve implanted.

Postoperative parameters included duration of mechanical ventilation, length of stay in the intensive care unit (ICU), total hospital stay, postoperative complications (stroke, renal insufficiency, bleeding requiring revision, atrial fibrillation, wound infection, conduction abnormalities, and low cardiac output), and in-hospital mortality.

Echocardiographic data

All patients underwent comprehensive transthoracic echocardiography. The following measurements were included: left ventricular ejection fraction (LVEF), pulmonary artery systolic pressure (PASP), indexed effective orifice area (EOAi), aortic annulus diameter, ascending aorta diameter, interventricular septal thickness, posterior wall thickness, end-diastolic and end-systolic diameters, and maximum and mean aortic transvalvular gradients.

Statistical analysis

Continuous variables are expressed as mean ± standard deviation and compared using the Student’s t-test. Categorical variables are presented as frequencies and percentages and compared using the chi-square test or Fisher’s exact test where appropriate. A p-value < 0.05 was considered statistically significant. All analyses were performed using IBM SPSS Statistics for Windows, Version 26.0 (IBM Corp., Armonk, NY).

Surgical technique

The surgical technique description is the same for both concomitant surgery and isolated aortic valve surgery. In both types of procedures, median sternotomy and ministernotomy were used, with the latter more commonly employed in cases of isolated aortic valve surgery. There were no procedural differences between the two surgical approaches.

The routine surgical approach was standard. After conventional monitoring and induction of general anesthesia, median sternotomy (or mini-sternotomy) was performed. The pericardium was opened, systemic heparinization was achieved, and the ascending aorta was cannulated. Venous drainage was via single right-atrium cannulation, and a left-heart vent was inserted through the right superior pulmonary vein. Once cardiopulmonary bypass was commenced, the aorta was cross-clamped. Cardioplegia was delivered antegrade via the aortic root and/or directly into the coronary ostia, with an initial crystalloid dose followed by blood cardioplegia.

The ascending aorta was resected, excising all diseased tissue. Pledgeted sutures were placed in the aortic annulus, and the mechanical or biological prosthesis parachuted into position. Thereafter, the prosthetic graft was anastomosed, with distal and proximal suture lines reinforced with Teflon felt as dictated by aortic-wall thickness. The heart was de-aired and the aorta unclamped. A temporary epicardial ventricular pacing wire, mediastinal drains, and chest closure with stainless-steel wires completed the procedure.

## Results

The detailed demographic characteristics, comorbidities, and preoperative functional status of the patients in both groups are summarized in Table 1. The cohort comprised 131 patients undergoing concomitant surgery and 491 patients undergoing isolated AVR. Age differed significantly (P < 0.001): mean 62.28 ± 10.76 years for isolated AVR versus 57.33 ± 11.90 years for concomitant surgery. Sex distribution was also significant (P < 0.001): men represented 77.9% (102 patients) of the concomitant group versus 63.3% (311 patients) of the isolated group.

**Table 1:** General data and comorbidities NYHA: New York Heart Association, COPD: chronic obstructive pulmonary disease. A p-value <0.05 was considered statistically significant. The Chi-square test and Fisher’s exact test were used.

Data	AVR (Nr/percentage)	AVR+AAR (Nr/percentage)	P-value
Male	311/63.3%	102/77.9%	<0.001
Female	180/36.7%	29/22.1%	
Elective surgery	447/91.0%	123/93.9%	0.828
Urgent surgery	43/8.8%	8/6.1%	
Emergent surgery	1/0.2%	0/0%	
NYHA I	4/0.8%	1/0.8%	<0.001
NYHA II	191/39.6%	62/47.3%	
NYHA III	266/55.2%	66/50.4%	
NYHA IV	21/4.4^	2/1.5%	
Arterial hypertension	350/71.6%	97/74%	<0.001
Kidney disease	12/2.5%	1/0.8%	0.105
Diabetes mellitus	85/17.4%	5/3.8%	<0.001
Peripheral arteropathy	8/1.6%	0/0%	0.287
Smoking	77/15.7%	17/13%	0.156
Obesity	12/2.5%	4/3.1%	0.601
COPD	18/3.7%	1/0.8%	0.229

A total of 43 patients (8.8%) underwent urgent isolated aortic valve replacement, while one patient underwent emergency surgery. By contrast, 447 patients (91.0%) underwent elective procedures. In the concomitant surgery group, eight patients (6.1%) underwent urgent intervention, whereas 123 patients (93.9%) underwent elective procedures. Surgical urgency was not a statistically significant variable.

The NYHA functional class was a statistically significant factor. In the isolated surgery group, four patients (0.8%) presented at admission with NYHA Class I, and in the concomitant group, only one patient presented in NYHA Class I. The majority of patients in both surgical groups were classified as NYHA II or III. In the isolated group, 457 patients (94.8%) were in NYHA II-III, while in the concomitant group, 128 patients (97.7%) were in NYHA II-III. It was observed that a higher number of patients undergoing isolated AVR were in the most advanced functional class, NYHA IV: 21 patients (4.4%) compared to two patients (1.5%) in the concomitant group.

The clinical relevance of NYHA class distribution reflects the severity of heart failure symptoms and provides a standardized measure of preoperative functional status. NYHA classification contributes to serving as a risk stratification tool; patients in higher NYHA classes (III-IV) generally have a worse prognosis and may experience higher perioperative risk or slower postoperative recovery, and help interpret outcome data (e.g., changes in functional status, survival, or complications) by providing a preoperative reference point. NYHA class distribution is not only descriptive but also essential for contextualizing clinical outcomes and assessing the effectiveness of the surgical interventions under study.

Regarding comorbidities in patients undergoing both surgical types, arterial hypertension and diabetes mellitus were statistically significant. Arterial hypertension was the most frequently encountered comorbidity in both groups: 350 patients (71.6%) undergoing isolated AVR had arterial hypertension, compared to 97 patients (74%) in the concomitant group. Diabetes mellitus, also statistically significant, was noted in 85 patients (17.4%) undergoing isolated AVR and in five patients (3.8%) in the concomitant group. There was a predominance of diabetes in the isolated AVR group.

Smoking was also considered a relevant risk factor, with 77 patients (15.7%) in the isolated group and 17 patients (13%) in the concomitant group identified as smokers. However, smoking was not statistically significant.

Other comorbid conditions assessed included renal pathology, peripheral artery disease, obesity, and COPD. None of these variables was found to be statistically significant.

Table 2 demonstrates that two other statistically significant factors were body surface area (BSA) and the indexed effective orifice area (EOAi), both with P < 0.001. BSA was lower in the isolated aortic valve replacement group (1.81 ± 0.16) compared to the concomitant surgery group (1.88 ± 0.19), whereas EOAi was higher in the concomitant surgery group (1.03 ± 0.27) than in the isolated aortic valve group (0.87 ± 0.22). Significant differences in BSA and EOAi may reflect variations in patient characteristics or anatomy, and their relevance lies in how they influence prosthesis selection, risk of prosthesis-patient mismatch (PPM), and postoperative hemodynamic performance.

**Table 2:** Echocardiography data BSA: body surface area, EOAi: indexed effective orifice area of the prosthesis, PsAP: pulmonary artery systolic pressure, ThPW: thickness posterior wall, ThS: thickness of the septum, TDD: telediastolic diameter, TSD: telesystolic diameter, Max Grad: maximal gradient transaortic valve, Mean Grad: mean gradient transaortic valve. Continuous variables are expressed as mean ± standard deviation and compared between groups using the independent samples t-test, A p-value <0.05 was considered statistically significant.

Echocardiography data	AVR (mean/standard deviation)	AVR + AAR (mean/standard deviation	P-value
BSA	1.81 ± 0.16	1.88 ± 0.19	<0.001
EOAi	0.87± 0.22	1.03 ± 0.27	<0.001
Ejection fraction (%)	60.10 ± 9.18	59.20 ± 9.08	0.342
PaSP (mmHg)	40.49 ± 15.37	40.21 ± 12.29	0.925
Aortic annulus (mm)	21.62 ± 1.92	23.71 ± 1.99	<0.001
ThPW (mm)	14.18 ± 9.83	12.93 ± 2.5	0.256
ThS (mm)	15.27 ± 8.32	14.31 ± 2.68	0.293
TDD (mm)	55.38 ± 9.96	57.31 ± 7.65	0.059
TSD (mm)	38.68 ± 8.70	39.62 ± 8.15	0.420
Max grad (mmHg)	85.51 ± 21.27	84.67 ± 28.53	0.829
_Mean grad (mmHg)	52.90 ± 14.16	49.30 ± 18.30	0.112
Diameter of the ascending aorta	38.23 ± 5.00	51.96 ± 6.47	<0.001

Echocardiographic data were also included in the statistical analysis in this study. LVEF and pulmonary systolic artery pressure (PSAP) were similar in both surgical groups, with no statistically significant differences. The aortic annulus diameter was a significant factor (P < 0.001), being statistically smaller in the isolated valve surgery group (21.62 ± 1.92 mm) compared to the concomitant surgery group (23.71 ± 1.99 mm). The diameter of the ascending aorta was also statistically significant, with a larger value in the concomitant group (51.96 ± 6.47 mm) compared to the isolated valve group (38.23 ± 5.00 mm). This finding is consistent with the indication for ascending aortic replacement, which is >50 mm in the case of tricuspid aortic valves and >45 mm in the case of bicuspid valves. Other echocardiographic parameters were not statistically significant. There was no morphology valve analysis on both groups. 

Two of the most important variables included in the statistical analysis were cardiopulmonary bypass (CPB) time and aortic cross-clamp time (shown in Table 3). Statistically significant differences were observed in the mean CPB time (83.85 ± 22.63 minutes vs. 111.09 ± 23.67 minutes; P < 0.001) and mean aortic cross-clamp time (65.22 ± 19.20 minutes vs. 89.56 ± 20.71 minutes; P < 0.001). As expected, both CPB and cross-clamp times were longer in the concomitant surgery group compared to isolated aortic valve surgery.

**Table 3:** Operative and postoperative data Min: minute, CPB: cardiopulmonary by-pass, ICU: intensive care unit. Variables were analyzed using the independent samples t-test. A p-value of <0.05 is considered significant.

Data	AVR (mean/standard deviation)	AVR+AAR (mean/standard deviation)	P-value
CPB time (min)	83.85 ± 22.63	111.09 ± 23.67	<0.001
Aortic cross clamp time( min)	65.22 ± 19.20	89.56 ± 20.71	<0.001
Stay in the ICU (hours)	59.59 ± 65.60	52.49 ± 33.97	0.232
Hospital stay (days)	9.15 ± 4.45	9.55 ± 4.93	0.376
Respiratory assistance (hours)	16.12 ± 42.16	10.48 ± 5.26	0.004

ICU stay hours were higher in the isolated surgery group (59.59 ± 65.60 hours) compared to the concomitant group (52.49 ± 33.97 hours), but the difference was not statistically significant. The shorter respiratory assistance time observed in the concomitant group remained consistent after excluding statistical outliers, supporting the clinical relevance of this finding as a potential indicator of better early postoperative recovery. The number of hospital days was also similar between groups, with no statistically significant difference. ICU stay and respiratory assistance times were paradoxically longer in the isolated aortic valve surgery group may be explained by several factors. One possibility is selection bias, where patients undergoing isolated procedures may have had higher preoperative risk profiles or comorbidities not fully captured by baseline variables. Another consideration is that patients selected for concomitant surgery were often treated electively or earlier in their disease course, potentially contributing to smoother postoperative trajectories. These hypotheses warrant further investigation. 

Table 4 presents postoperative complications, in-hospital mortality, and the statistical analysis of these variables. Among postoperative complications, low cardiac output was statistically significant with a p-value < 0.001, whereas stroke, renal failure, postoperative bleeding, new-onset atrial fibrillation, wound infection, and conduction disturbances were not statistically significant. Low cardiac output showed a significant difference between the two groups, occurring in 18 patients (3.7%) in the isolated surgery group and two patients (1.5%) in the concomitant surgery group. Stroke, renal failure, bleeding, and conduction disturbances showed similar values between the two groups but without statistical significance. New-onset atrial fibrillation was the most frequently reported complication, with similar values between the two groups: 76 patients (15.5%) in the isolated group and 19 patients (14.5%) in the concomitant group.

**Table 4:** Postoperative complications Postoperative categorical outcomes were compared using the Chi-square test or the Fisher’s exact test when expected cell frequencies were less than 5. A p-value <0.05 is considered statistically significant.

Operative data	AVR (number of patients/percentage)	AVR+AAR (number of patients/percentage)	P-value
Hospital mortality	8/1.6%	3/2.29%	<0.001
Low cardiac output	18/3.7%	2/1.5%	<0.001
Stroke	5/1.0%	1/0.8%	0.955
Renal Insufficiency	1/0.2%	1/0.8%	0.486
Revision for bleeding	6/1.2%	1/0.8%	0.003
New atrial fibrillation	76/15.5%	19/14.5%	0.124
Wound infection	8/1.6%	2/1.5%	0.997
Conduction abnormalities	32/6.5%	9/6.5%	0.916

In-hospital mortality was statistically significant with a p-value < 0.001. In the isolated aortic valve replacement group, eight patients (1.6%) died, whereas in the concomitant group (aortic valve and ascending aorta replacement), three patients (2.29%) died.

## Discussion

Diseases affecting the aortic valve and ascending aortic aneurysm often have different etiologies. Intrinsic genetic and physiological abnormalities of the aortic wall are recognized causes of ascending aorta dilation (e.g., Marfan syndrome, bicuspid aortic valve). Another important factor is the hemodynamic pattern, where abnormal mechanical forces cause post-stenotic dilation of the ascending aorta. Furthermore, the aneurysm itself may result in valvular insufficiency by reducing the coaptation surface of the cusps. In cases where both diseases coexist (aortic valve disease and ascending aortic aneurysm), the surgical approach remains a topic of debate to this day: aggressive or conservative?

Between 2007 and 2023, our hospital center “Mother Teresa” treated 491 patients with isolated aortic valve replacement/repair and 131 patients with concomitant replacement/repair of the aortic valve and ascending aorta. In our country, this is the only study with such a patient series, and a review of the international literature revealed that our patient cohort is considerable. Brigham and Women’s Hospital, Boston [14] reported the largest series in the literature with 683 patients undergoing isolated surgery and 618 undergoing concomitant surgery. Lee et al. in Seoul [7] reported a series of 362 patients for isolated surgery and 70 patients for concomitant surgery. Other studies by Winkler et al. [15], Peterss et al. [16], and Lim et al. [17] reported patient series similar in size to our study.

The mean patient age was statistically significant (P < 0.001), with patients undergoing concomitant surgery being noticeably younger (57.33 ± 11.90 years) than those undergoing isolated surgery (62.28 ± 10.76 years). Kaneko et al. [14], in the largest published series, also reported that patients undergoing concomitant surgery were younger, and age was a statistically significant factor. Meanwhile, Lee et al. [7], Peterss et al. [16], and Lim et al. [17] reported no age differences between patients undergoing the two surgical types.

In our study, gender was statistically significant (P < 0.001) between the two surgical types. Males predominated in both surgery types (311 patients / 63.3% for AVR and 102 patients / 77.9% for AVR+AAR) compared to females (180 patients / 36.7% for AVR, 29 patients / 22.1% for AVR+AAR). Males predominated in almost all published studies, with proportions ranging from 55% to 68% (14,15,16,17,18). Gender was not statistically significant in any of the reviewed studies [14-18].

Surgical urgency showed no statistical difference (p = 0.828), despite 43 (8.8%) patients undergoing urgent isolated valve replacement and 1 (0.2%) patient undergoing emergency surgery, while eight (6.1%) patients underwent urgent concomitant surgery. In the literature, surgical urgency was not statistically significant [14-15]. Kaneko et al. [14] reported higher values for urgent surgery: 131 (19%) patients in isolated surgery and 87 (14%) in concomitant surgery, whereas Winkler et al. [15] reported no urgent cardiac surgeries.

NYHA class is a very important preoperative factor that significantly increases operative risk. In our study, the NYHA class was statistically significant. The majority of patients in both surgical types were classified as NYHA II and III. In isolated surgery, 457 (94.8%) patients were NYHA II-III, while in concomitant surgery, 128 (97.7%) patients were NYHA II-III. More patients undergoing isolated surgery were in the advanced NYHA IV class: 21 (4.4%) compared to only two (1.5%) in the concomitant group. Kaneko et al. [14] and Winkler et al. [15] presented NYHA as a statistically non-significant factor with p = 0.002, reporting 21 (7%) NYHA IV patients in isolated surgery and 14 (5%) in concomitant surgery, results similar to ours. Although statistical significance was noted in NYHA class distribution between groups, the proportions were clinically similar. This discrepancy may be influenced by referral bias or differences in surgical indication strategies, where patients with less symptomatic presentation (NYHA I-II) may have been referred for concomitant surgery as part of planned multi-valve or combined procedures, while those with more advanced symptoms were more likely to undergo isolated aortic valve surgery due to urgency or disease severity

Arterial hypertension and diabetes mellitus were statistically significant preoperative morbidities, whereas renal failure, peripheral artery disease, obesity, smoking, and COPD were not. In the largest patient study, Kaneko et al. [14] reported diabetes mellitus, dyslipidemia, and peripheral vascular disease as statistically significant preoperative morbidities (p < 0.001), while Winkler et al. [15] reported hypertension, diabetes mellitus, and dyslipidemia as significant. Lee et al. [7], with a comparable number of patients, reported no statistically significant morbidities.

Hypertension was the most common comorbidity in both surgery types: 350 (71.6%) patients in isolated AVR and 97 (74%) in concomitant AVR. Winkler et al. [15] reported hypertension as statistically significant in 637 (92.6%) patients undergoing isolated surgery and 24 (60.0%) in the concomitant group. In our study, hypertension occurred at similar rates in both groups, whereas Winkler et al. [15] noted a predominance of hypertension in isolated surgery. Lee et al. [7] reported much lower hypertension values than ours: 81 (22.4%) patients in isolated surgery and 11 (15.7%) in concomitant surgery.

Diabetes mellitus, a statistically significant factor, was found in 85 (17.4%) isolated surgery patients and five (3.8%) concomitant surgery patients. A predominance of diabetes in isolated AVR was observed, a finding also reported by Kaneko et al. [14], with 111 (16%) isolated surgery patients and 41 (7%) in concomitant surgery. Winkler et al. [16] similarly reported diabetes as a statistically significant factor, predominating in isolated surgery: 184 (26.7%) versus 1 (2.5%).

Smoking also occupied an important place among risk factors, with 77 (15.7%) patients in isolated surgery and 17 (3%) in concomitant surgery, although it was not statistically significant. Kaneko et al. [14] and Winkler et al. [16] reported smoking as not statistically significant, with prevalence ranging between 32% and 35% in isolated surgery and 31% and 42% in concomitant surgery.

BSA and SEPi are very important factors in aortic valve surgery. In our study, BSA was a statistically significant factor (P < 0.001), being lower (1.81 ± 0.16) in isolated valve surgery compared to concomitant surgery (1.88 ± 0.19). SEPi was also statistically significant (P < 0.001), being higher in the concomitant group (1.03 ± 0.27) than in the isolated group (0.87 ± 0.22).

In the statistical analysis of echocardiographic data, aortic annulus diameter and ascending aorta dimensions were statistically significant (P < 0.001), whereas ejection fraction, PSAP, TS, TMP, DTD, DTS, and average and maximum transvalvular gradients were not. The annulus diameter was significantly larger in the concomitant group (23.71 ± 1.99 mm) compared to isolated AVR (21.62 ± 1.92 mm). The diameter of the ascending aorta was another statistically significant factor. In the concomitant group, the ascending aorta was predictably larger (51.96 ± 6.47 mm), as also reported by Kaneko et al. [14], who found a statistically significant value (47.3 ± 4.7 mm). Peterss et al. [16] also found ascending aorta diameter to be statistically significant (P < 0.001), reporting values of 48.5 ± 7.6 mm in concomitant surgery and 34.8 ± 4.4 mm in isolated AVR.

From the operative and postoperative data, CPB time and aortic clamp time were statistically significant (P < 0.001), whereas respiratory assistance, ICU stay, and hospital stay were not. Mean CPB time was significantly longer in concomitant surgery (111.09 ± 23.67 min) than in isolated surgery (83.85 ± 22.63 min). Most literature sources report CPB time as statistically significant [14-18]. Peterss et al. [16] reported it as statistically significant: 144.1 ± 25.7 minutes in concomitant surgery and 91.1 ± 14.4 min in isolated surgery. Winkler et al. [7] also reported it as statistically significant: 118.5 (104-131) minutes in concomitant surgery and 91 (74.5-108.5) minutes in isolated surgery - values similar to ours.

Aortic clamp time was also statistically significant. In isolated AVR, clamp time was lower (65.22 ± 19.20 minutes) compared to concomitant surgery (89.56 ± 20.71 minutes). The literature also reports clamp time as statistically significant, with values ranging between 84 and 99 min for concomitant and 63-68 min for isolated surgery [15- 17]. Our operative times are comparable with those in the literature and suggest that this surgery can be safely performed by our surgical teams with excellent postoperative outcomes. ICU hours, hospital stay, and ventilatory support were not statistically significant.Although CPB and cross-clamp times were longer in the concomitant surgery group, this did not translate into statistically significant differences in major postoperative complications or ICU stay. Nonetheless, prolonged operative times are well-recognized risk factors for adverse outcomes such as bleeding, low cardiac output, prolonged ventilation, and infection. The absence of significant differences in our cohort may reflect effective intraoperative management, meticulous surgical technique, and optimized perioperative protocols that mitigated the potential impact of extended operative duration. However, these findings should be interpreted cautiously, as even non-significant trends may carry clinical relevance, particularly in high-risk patients.

In our statistical analysis, low cardiac output was the only postoperative complication that was statistically significant (p < 0.001), whereas stroke, renal failure, postoperative bleeding, new-onset atrial fibrillation, wound infection, and conduction disturbances were not. A literature review [7,15,16] confirmed that none of these complications were statistically significant. In our study, low cardiac output was significantly different between groups: 18 patients (3.7%) in isolated surgery and two patients (1.5%) in concomitant surgery. Interestingly, the incidence of low cardiac output was statistically lower in the concomitant surgery group, despite this group being considered higher risk due to the complexity of the procedure. This counterintuitive finding may be partly explained by selection bias, where patients selected for concomitant surgery were more thoroughly pre-evaluated or operated on electively. In addition, differences in perioperative management strategies, such as intraoperative myocardial protection or more aggressive hemodynamic monitoring and support, may have contributed to better immediate cardiac function post-surgery in this group. These factors highlight the importance of individualized surgical planning and multidisciplinary coordination in influencing early postoperative outcomes. Peterss et al. [16] reported very low rates of low output - three patients (4%) in both groups.

New-onset atrial fibrillation is reported as the most frequent complication in nearly all literature sources, with rates ranging between 11% and 33% in isolated and 9% and 42% in concomitant surgery [14-17]. In our study, there was no statistical difference between the two groups: 76 patients (15.5%) in isolated and 19 patients (14.5%) in concomitant surgery. Stroke occurred at favorable rates: five patients (1.0%) in isolated and one patient (0.8%) in concomitant surgery, in line with literature reporting a 1% incidence [7,15,16]. Several postoperative outcomes, including atrial fibrillation, stroke, and ICU stay, did not show statistically significant differences between the isolated and concomitant surgery groups. This absence of difference may reflect consistent and effective surgical and perioperative management across both groups, suggesting that the addition of concomitant procedures did not adversely impact these specific outcomes. However, it is also important to consider the possibility of limited statistical power to detect small but potentially meaningful differences, especially for less frequent complications such as stroke. 

Conduction disturbances are a key complication in aortic surgery. In our study, this was not statistically significant, affecting 6% of both populations. Winkler et al. [15] reported pacemaker implantation more frequently in concomitant surgery (10.0% vs 5.4%), whereas Peterss et al. [16] reported the opposite (12% in isolated, 5% in concomitant).

In-hospital mortality in our study was statistically significant between groups (p < 0.001): eight patients (1.6%) in isolated AVR versus three patients (2.29%) in concomitant AVR. The slightly higher mortality in concomitant surgery reflects its inherently higher operative risk. Although the difference in mortality between groups reached statistical significance (1.6% vs. 2.29%), the absolute difference is minimal and may lack clinical relevance. This small variation is unlikely to impact clinical decision-making, particularly given the low overall mortality in both groups. Kaneko et al. [14], in the largest series (683 AVR vs. 618 AVR+AAR), found in-hospital mortality to be statistically nonsignificant but reported slightly higher mortality in isolated surgery (0.7%, five patients) compared to concomitant (0.3%, two patients). Similarly, Lee et al. [7], Winkler et al. [15], Peterss et al. [16], and Lim et al. [17] reported hospital mortality as statistically non-significant. Paradoxically, these studies [7,15,16] reported higher mortality in isolated AVR. Winkler et al. [15] reported zero mortality in concomitant surgery and 3.3% (four patients) in isolated surgery, although only 40 cases of concomitant surgery were included compared to 131 in our study. Lee et al. [7] reported 0% mortality in 70 concomitant cases and 1.2% (six patients) in isolated AVR - values close to ours.

Recent guideline updates clearly define indications for replacing the ascending aorta in patients undergoing AVR, particularly in the presence of a bicuspid aortic valve [8]. In clinical routine, patients with a bicuspid aortic valve require special attention. Elective ascending aortic replacement, as a concomitant procedure, is performed prophylactically and aims to prevent major aortic events. Therefore, it is essential to assess the risk of aortic diameter progression.

Girdauskas et al. [18] analyzed 325 patients with tricuspid and bicuspid aortic valves who underwent isolated AVR for aortic stenosis and concurrent ascending aortic dilation. Reintervention rates due to aneurysm progression were low (3-5%), and aortic dissection occurred only in tricuspid valve patients. Similar findings were published by Longi et al. [19] in a cohort of patients with bicuspid valve and ascending aorta dilation (40-45 mm): among 431 patients, only two underwent reintervention, leading the authors to conclude that mild dilation does not justify aortic replacement. 

The decision threshold for ascending aortic replacement remains an area of active debate, especially in patients with bicuspid aortic valves (BAV). While current guidelines [8] often recommend replacement at a diameter of ≥50 mm in the absence of additional risk factors, some experts advocate for a lower threshold of 45 mm, particularly when concomitant valve surgery is planned or when additional risk factors (e.g., family history, rapid growth, or asymmetric dilation) are present. In the context of BAV, where aortic wall pathology may predispose to earlier dissection or rupture, some centers adopt a more aggressive approach, replacing the ascending aorta at ≥45 mm, even in the absence of other criteria. This practice reflects a precautionary philosophy but must be weighed against the risks of more extensive surgery. Our study cohort reflects this ongoing variability in surgical thresholds, underscoring the need for individualized decision-making and further research to refine size-based criteria

Considering the above findings and literature review, the surgical approach to concomitant AVR and ascending aortic replacement should always be guided by current guidelines and the surgical team's experience. Surgeon experience likely plays a role in outcomes, particularly in the concomitant surgery group where procedural complexity is higher. Variability in technical expertise, decision-making, and intraoperative judgment can significantly impact both operative efficiency and complication rates. Although our study did not specifically control for surgeon volume or experience, it is important to recognize that operator skill and institutional familiarity with complex procedures. With the advent of minimally invasive aortic valve surgery via mini-sternotomy, ascending aortic replacement can certainly be added to the surgical plan. Although the risk of reintervention and fatal events associated with non-operated but dilated ascending aorta is relatively low, it is our duty to provide the most optimal and durable solution for all patients.

Limitations

This study is subject to several limitations. First, its retrospective nature may introduce selection bias and limit the ability to infer causality. Second, being a single-center study, the findings may not be generalizable to other institutions with different surgical techniques, patient demographics, or postoperative care protocols. Third, although statistical adjustments were performed, unmeasured confounding variables may still affect the outcomes. Finally, long-term follow-up data, including reintervention rates and survival analysis, were not available and should be addressed in future prospective studies.

## Conclusions

Concomitant replacement of the aortic valve and ascending aorta significantly increases cardiopulmonary bypass and aortic clamp times compared to isolated AVR. In-hospital mortality in our study was statistically significant but remains comparable with many international centers. Our findings suggest that concomitant surgery involving the aortic valve and ascending aorta is safe and yields comparable in-hospital outcomes to isolated aortic valve surgery. This conclusion is limited to short-term, in-hospital results and does not account for mid- or long-term outcomes. Importantly, the results underscore that careful patient selection remains a critical factor in achieving favorable early outcomes, particularly when managing complex or combined procedures. The above postoperative results further confirm that concomitant surgery of the ascending aorta and aortic valve, as performed by the Cardiac Surgery Service at our University Hospital Center, is a safe surgical approach with outcomes very comparable to those reported in the literature. 
